# Microscopic study of electrical properties of CrSi_2 _nanocrystals in silicon

**DOI:** 10.1186/1556-276X-6-209

**Published:** 2011-03-09

**Authors:** László Dózsa, Štefan Lányi, Vito Raineri, Filippo Giannazzo, Nikolay Gennadevich Galkin

**Affiliations:** 1 Research Institute for Technical Physics and Materials Science, P. O. Box 49, H-1525 Budapest, Hungary; 2 Institute of Physics of the Slovakian Academy of Sciences, Dúbravská Cesta 9, SK-854 11 Bratislava, Slovakia; 3 CNR-IMM, Strada VIII 5, 95121Catania, Italy; 4 Institute for Automation and Control Processes of Far Eastern Branch of Russian Academy of Sciences, 690041 Vladivostok Radio 5, Russia

## Abstract

Semiconducting CrSi_2 _nanocrystallites (NCs) were grown by reactive deposition epitaxy of Cr onto *n*-type silicon and covered with a 50-nm epitaxial silicon cap. Two types of samples were investigated: in one of them, the NCs were localized near the deposition depth, and in the other they migrated near the surface. The electrical characteristics were investigated in Schottky junctions by current-voltage and capacitance-voltage measurements. Atomic force microscopy (AFM), conductive AFM and scanning probe capacitance microscopy (SCM) were applied to reveal morphology and local electrical properties. The scanning probe methods yielded specific information, and tapping-mode AFM has shown up to 13-nm-high large-area protrusions not seen in the contact-mode AFM. The electrical interaction of the vibrating scanning tip results in virtual deformation of the surface. SCM has revealed NCs deep below the surface not seen by AFM. The electrically active probe yielded significantly better spatial resolution than AFM. The conductive AFM measurements have shown that the Cr-related point defects near the surface are responsible for the leakage of the macroscopic Schottky junctions, and also that NCs near the surface are sensitive to the mechanical and electrical stress induced by the scanning probe.

## Introduction

Chromium disilicide (CrSi_2_) is a narrow band semiconductor (*E*_g _= 0.35 eV [1]), which can be epitaxially grown on Si (111) [2]. Strong increase of hole mobility and decrease of hole concentration have been observed in CrSi_2 _epitaxial films on Si(111) [3] that corresponds to considerable alterations in their band structure. In previous studies of Cr deposition on Si(111) the formation of self-organized semiconductor CrSi_2 _islands has been observed by differential optical spectroscopy (DOS) and the threshold for 3D nanosize island formation has been determined [4]. The MBE growth of silicon cap over the CrSi_2 _islands was found to be optimal at 700°C, with 50-nm Si cap thickness [4]. Under these conditions silicon-silicide heterostructures with CrSi_2 _nanocrystallites (NCs) have been grown from 0.6-nm Cr deposited onto 550°C silicon [4]. The electrical characteristics were measured in 400 μm × 400 μm Schottky junctions. Optical properties of the samples were studied in an ultrahigh vacuum (UHV) chamber "VARIAN" with a base pressure of 2 × 10^-8 ^Pa equipped with AES and DOS [5] facilities. A new migration mechanism of the CrSi_2 _NCs was found, the NCs are transferred through nanopipes [6], which results in CrSi_2 _NCs with different depth distributions. Macroscopic Schottky junctions include large number of NCs in different sizes and depths, and therefore, to understand the behaviour of the devices, the electrical parameters of single NCs are needed.

In this study, CrSi_2 _NCs, covered with 50-nm epitaxial silicon but having different depth distributions, were investigated. In order to improve the electrical characterization of these nanostructures, the electrical parameters obtained by scanning probe tip are compared with electrical characteristics measured in macroscopic Schottky devices.

## Experimental

The CrSi_2 _NCs and the silicon cap layer were grown in UHV chamber without breaking the vacuum. Samples were cut from *n*-type 7.5 Ωcm Si (111) substrates. The silicon was cleaned by annealing at 700°C for 4-5 h, cooling during 12 h, and cleaning flashes were applied at 1250°C. Surface purity was controlled *in situ *by AES. Cr was reactive epitaxy deposited on 550°C substrate from a Tantalum tube. The Cr deposition rate was about 0.02-0.04 nm/min controlled by a quartz sensor. 50 nm silicon cap was grown by MBE at 700°C at deposition rate of 3-4 nm/min over the NCs.

The morphology was studied by atomic force microscopy (AFM) in contact and tapping-mode. Conductive AFM and scanning probe capacitance microscopy (SCM) were measured in contact-mode using a Pt-coated Si tip and a diamond-coated Si tip, respectively. For the SPM characterisations a VEECO Dimension V microscope was used.

Schottky junctions were prepared by evaporation of 400 μm × 400 μm square gold dots onto the silicon. Gallium was scratched on the back side to form ohmic contact. The depth distribution of the NCs was measured by transmission electron microscopy (XTEM) using the sample preparation method described in [7].

## Results and discussion

XTEM measurements have shown that the CrSi_2 _NCs migrate towards the surface during the cap growth [6,8]. The depth distribution of the NCs was different depending on the deposition rate. Two types of samples were investigated. In one type, most of the NCs were seen by XTEM near the deposition depth, while in the other type they were observed mostly near the surface [6].

### Electrical characteristics

**Figure 1:**
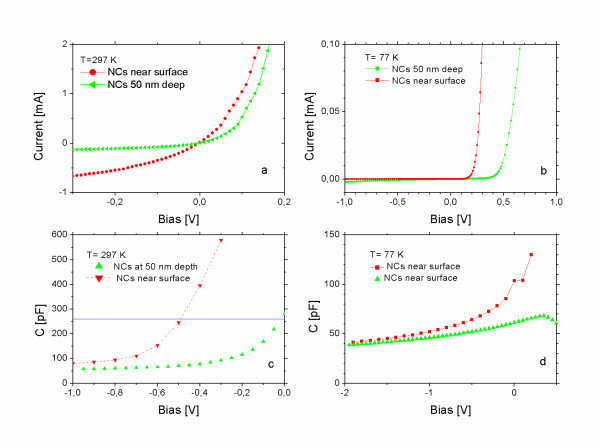
**Characteristics of the Schottky junctions on Si/CrSi_2 _NC/Si structures: ***I*-*V *measured at room temperature **(a) **and at 77 K **(b)**; *C*-*V *measured at room temperature **(c) **and at 77 K **(d)**.

**Figure 2:**
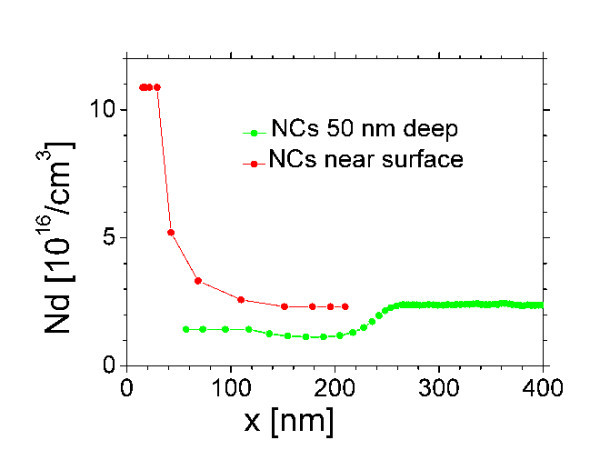
The apparent donor-concentration profiles in the two types of samples calculated from *C*-*V *characteristics measured at different temperatures.

Typical current-voltage (*I*-*V*) characteristics in Schottky junctions of the two different types measured at 297 K are shown in Figure 1a, and those measured at 77 K are shown in Figure 1b. The series resistance dominates the forward, and leakage resistance dominates the reverse *I*-*V *characteristics in samples where the NCs migrated near the silicon surface. The typical leakage resistance is about 1 kΩ at 297 K and increases to 56 kΩ at 77 K. The cited resistance values are not related to the figures. The figures demonstrate the different types of *I*-*V*s. The leakage resistance is thermally activated, indicating that the Fermi level in the cap is pinned by point defects at about 160 meV from the conduction band. The thermal activation of the leakage resistance was evaluated by linear fits to the reverse *I*-*V *and by plotting the fitted resistance values as a function of reciprocal temperature. The capacitance-voltage (*C*-*V*) characteristics of the junctions measured at room temperature are shown in Figure 1c, and those measured at 77 K are shown in Figure 1d. Schottky junction capacitance 260 pF--indicated as a line in Figure 1c--corresponds to 50-nm depleted layer thickness, equal to the nominal cap thickness. Below -1 V reverse bias at low temperature, the doping calculated from the 1/*C*^2^-voltage plot is appropriate for the semiconductor substrate in both types of samples. The doping concentration profile was calculated from the *C*-*V *characteristics measured at different temperatures (not shown in the figures). The calculated doping profiles in the two type of samples are shown in Figure 2. The samples with NCs migrating near the surface show high concentration of donors, while in samples with NCs remaining near 50-nm deposition depth, the donor concentration is low.

### DLTS characterization

The DLTS spectra were measured at -1 V reverse bias, and 20 μs, 0 V filling pulses. The energy position of the deep level calculated from the DLTS Arrhenius plot is about 0.25 eV, appropriate for the Cr level at *E*_c_--0.27 eV in *n*-type silicon [9]. The large concentration of doping depicted in Figure 2 in samples where the NCs migrated near the surface is explained by large concentration of Cr-related point defects in the cap. In the samples with NCs near the deposition depth, the low donor concentration depicted in Figure 2 is explained by the low concentration of Cr-related deep-level defects. The markedly different concentrations of Cr-related point defect in the two types of samples indicate that these defects may be related to the observed migration of the NCs during the cap growth. To enable us understand the role of the Cr-related defects in migration of NCs, we require further experiments.

### AFM measurements

**Figure 3:**
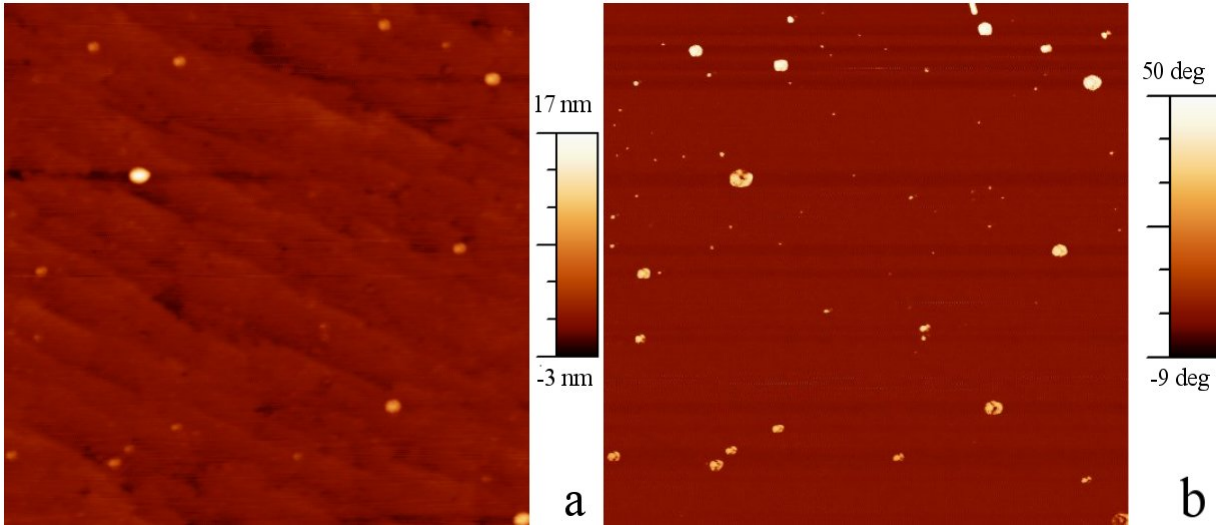
**Tapping-mode AFM images of a 1 μm × 1 μm area on the sample with NCs below the 50-nm silicon cap**: **(a) **amplitude, **(b) **phase.

Tapping-mode AFM amplitude and phase images of the samples with NCs near the surface are shown in Figure 3a,b, respectively. The tapping-mode AFM amplitude (Figure 3a) is not sensitive to the CrSi_2 _NCs. Several bigger NCs appear in the phase image (Figure 3b). We suppose that it is due to the electrical interaction of NCs with the vibrating scanning tip. The phase of the vibration changes, but does not cause energy dissipation, interpreted as height in the amplitude image. Some spherical protrusions appear with a diameter of about 90 nm and a height of 12 nm in the amplitude. The morphology measured in contact-mode does not show these large protrusions. The difference can be an effect of the pressure of the tip in contact-mode and the possible wear of the sample, since repeated scans over the imaged areas has shown visible degradation of the surface. However, we suppose that these protrusions are mainly due to areas with large NC density in the cap, resulting in virtual height increase in tapping-mode amplitude image.

**Figure 4:**
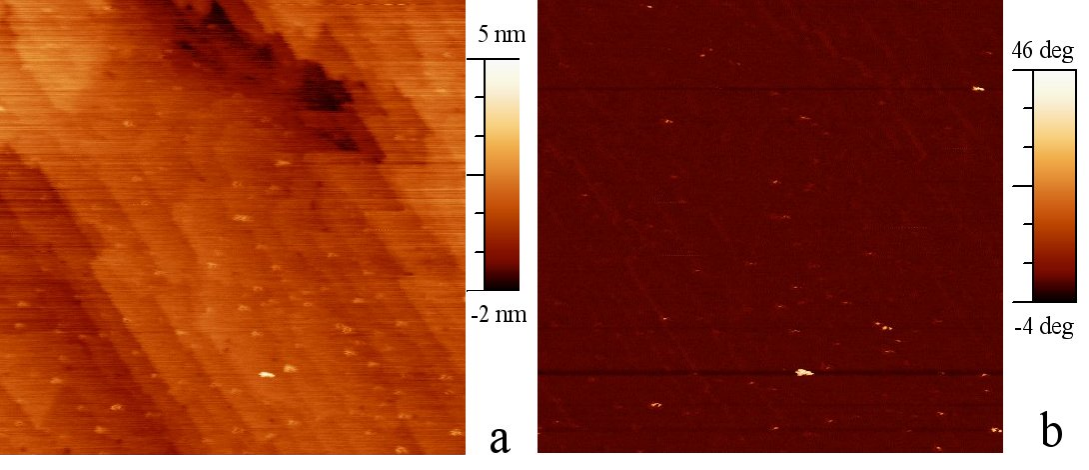
**Tapping-mode images of a 1 μm × 1 μm area on the sample with NCs near the surface**. **(a)** amplitude, **(b)** phase.

In samples with NCs 50 nm deep below the surface, the morphology and the phase of the tapping-mode AFM of the silicon surface measured are shown in Figure 4a,b, respectively. The NCs are hardly visible in both amplitude and phase images; the interaction of the vibrating tip with NCs embedded 50 nm deep in silicon is weak.

### Conductive AFM measurements

The sample with NCs close to the surface exhibited large leakage at room temperature in macroscopic junctions. To understand the reason of leakage this sample was analysed by conductive AFM. The conductive tip is scanned on the surface, and the current at a given voltage is recorded and mapped. Conductive AFM reveals the local conductivity differences in the vicinity of NCs. Across most of the surface, the current was nearly constant and even independent of the polarity of the bias. Locally, evidence of rectification could be observed. The tip-wafer junction can be easily degraded by the local current load, and so the reliability of repeated measurements using other bias conditions is questionable. The results show that primarily the large concentration of Cr-related point defect in this sample is responsible for the leakage. The large local electric field around the NCs may also act as local short-circuit path; however, this kind of leakage was not strongly dependent on temperature, as observed in large-area Schottky junctions.

### SCM measurements

**Figure 5:**
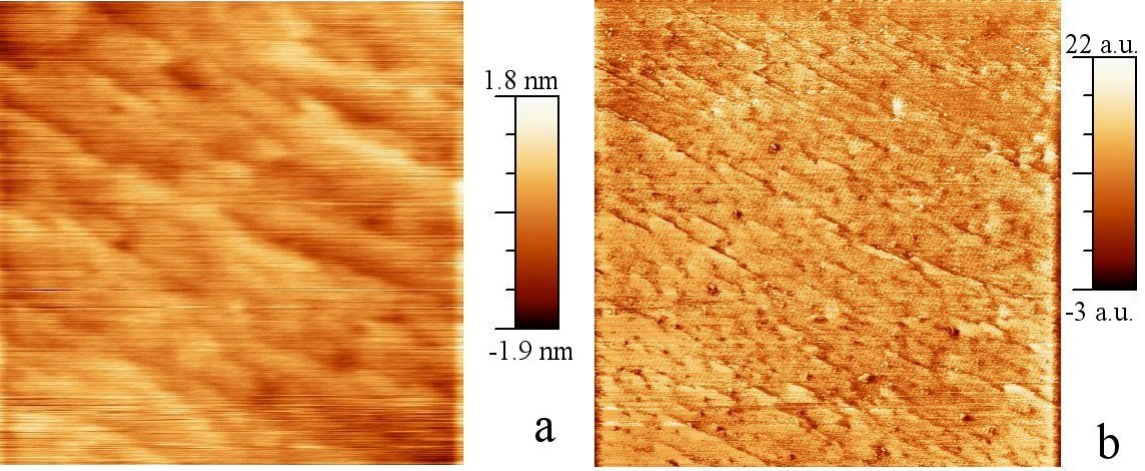
**Contact-mode scanning probe images of a 1 μm × 1 μm area on the sample with NCs near the surface**. **(a)**. AFM amplitude image. **(b)**. SCM image of the same area.

The contact-mode AFM amplitude and SCM images recorded simultaneously in sample with redistributed NCs are shown in Figure 5a,b, respectively. The SCM shows definitely better contrast and spatial resolution than AFM, indicating that the detection of NCs is improved when electrical interaction is involved in the image. The size of the observed objects is appropriate for NC sizes seen earlier in XTEM and in AFM images [6,8].

**Figure 6:**
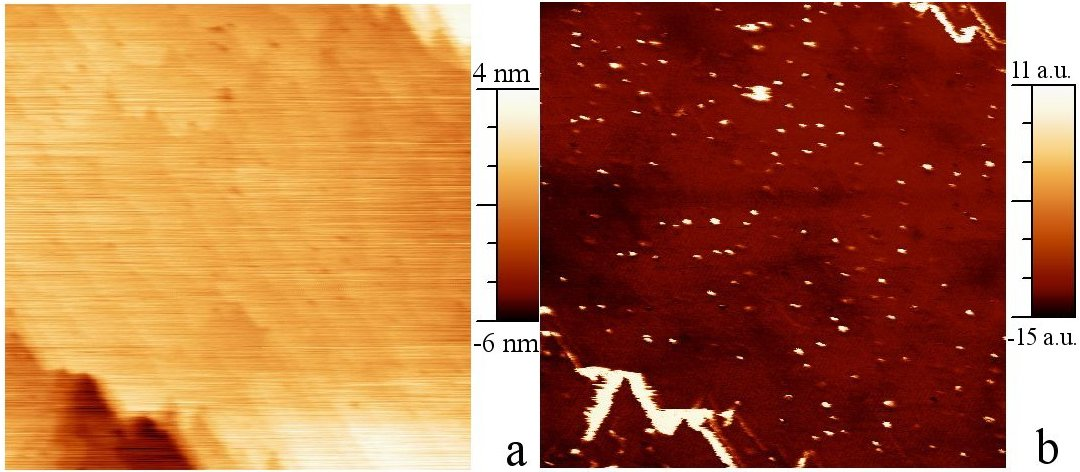
**Contact-mode scanning probe images of a 1 μm × 1 μm area on the samples with NCs 50 nm below the surface**. **(a)**. AFM amplitude image. **(b)**. SCM image of the same area.

**Figure 7:**
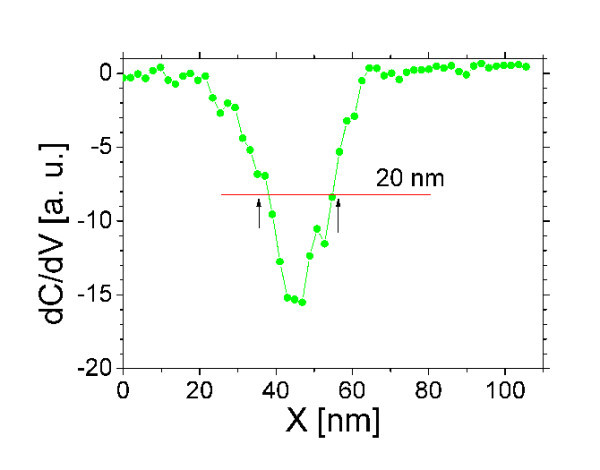
An SCM line profile across a NC 50 nm below the surface.

The contact-mode AFM and SCM images of the sample with NCs at 50-nm depth are shown in Figure 6a,b, respectively. The NCs are hardly visible in AFM, as the sample surface is flat. The NCs are apparent in SCM. The higher conductivity of inclusions increases the locally sensed capacitance, and thus, the difference of electrical properties of silicon and CrSi_2 _gives a better contrast for the detection of the NCs by scanning tip capacitance sensing. NCs deep below the surface are revealed in SCM images, and are not shown in the morphology measured simultaneously. Deep NC features are generally expected to appear somewhat smeared [10]. A cross section of the SCM image across a NC is shown in Figure 7. The half-width of the peak agrees with the size of the NCs. It shows that the interaction of the charged NC and the measuring tip is strong, which controls the transport, and we assume that the measured capacitance is dominated by the NC-host junction. The measurements indicate that the NCs embedded deep--having electrical characteristics different from the defect-free host--can be detected using the SCM with high resolution.

## Conclusions

Electrical characteristics of monolithic Si/CrSi_2 _NCs/Si structures with different depth distributions of the NCs were investigated in large-area Schottky junctions by *I*-*V *and *C*-*V *measurements, and locally by scanning probe techniques, conductive AFM and SCM. It is shown that the CrSi_2 _NCs in 50-nm depth in a defect-free silicon matrix can be detected by electrically active probes with a resolution comparable to the NC size, and that the SCM gives better contrast and spatial resolution than the tapping-mode AFM. We suppose that this is because the charged NCs control the electric transport. It shows that in appropriate host crystal, SCM may reveal the individual NC-host junction properties. Tapping-mode AFM image is distorted by the interaction of the NCs with the vibrating tip. It is shown that high concentration of Cr-related defects induces leakage in large area Schottky junctions. The results show that the measuring tip-wafer current may seriously degrade the devices with NCs near the surface.

## Abbreviations

AFM: atomic force microscopy; DOS: differential optical spectroscopy; NCs: nanocrystallites; SCM: scanning probe capacitance microscopy; UHV: ultrahigh vacuum; XTEM: transmission electron microscopy.

## Competing interests

The authors declare that they have no competing interests.

## Authors' contributions

LD designed the study, carried out the electrical measurements on Schottky junctions, and drafted the manuscript, SL, VR, and FG measured the scanning probe measurements, NG has prepared the samples. All authors read and approved the final manuscript.
